# Glomerular filtration in the aging population

**DOI:** 10.3389/fmed.2022.769329

**Published:** 2022-09-15

**Authors:** Irene L. Noronha, Guilherme P. Santa-Catharina, Lucia Andrade, Venceslau A. Coelho, Wilson Jacob-Filho, Rosilene M. Elias

**Affiliations:** ^1^Renal Division, Hospital das Clinicas, University of São Paulo Medical School, São Paulo, Brazil; ^2^Laboratory of Cellular, Genetic and Molecular Nephrology, University of São Paulo Medical School, São Paulo, Brazil; ^3^Geriatric Division, Hospital das Clinicas, University of São Paulo Medical School, São Paulo, Brazil

**Keywords:** glomerular filtration rate (GFR), elderly, renal function, senescence, aging, estimated GFR

## Abstract

In the last decades, improvements in the average life expectancy in the world population have been associated with a significant increase in the proportion of elderly people, in parallel with a higher prevalence of non-communicable diseases, such as hypertension and diabetes. As the kidney is a common target organ of a variety of diseases, an adequate evaluation of renal function in the approach of this population is of special relevance. It is also known that the kidneys undergo aging-related changes expressed by a decline in the glomerular filtration rate (GFR), reflecting the loss of kidney function, either by a natural senescence process associated with healthy aging or by the length of exposure to diseases with potential kidney damage. Accurate assessment of renal function in the older population is of particular importance to evaluate the degree of kidney function loss, enabling tailored therapeutic interventions. The present review addresses a relevant topic, which is the effects of aging on renal function. In order to do that, we analyze and discuss age-related structural and functional changes. The text also examines the different options for evaluating GFR, from the use of direct methods to the implementation of several estimating equations. Finally, this manuscript supports clinicians in the interpretation of GFR changes associated with age and the management of the older patients with decreased kidney function.

## Introduction

The proportion of older people in the population is increasing worldwide, reflecting profound demographics changes overall. In 2020, there were 727 million elderly individuals aged 65 years or over, representing 9.3% of the world's population—a number expected to double by 2050 (1). As a result of the increase in life expectancy, non-communicable diseases such as diabetes and hypertension have become more prevalent, with a consequent increase in the prevalence of chronic kidney disease (CKD).

Over the years, the kidneys undergo aging-related changes expressed by a decline in the glomerular filtration rate (GFR), reflecting the loss of kidney function. Anatomical and functional changes become more evident in advanced ages, accounting for the GFR decrease. The loss of kidney function with advancing age indicates a physiological decline in GFR associated with healthy aging as a natural senescence process (2), a condition difficult to distinguish from the pathological decline in GFR related to kidney diseases. Half of adults over 70 years have a GFR, either measured or estimated, of <60 mL/min/1.73 m^2^ (3). However, it is still not clear whether a reduction in GFR is a consequence of the aging process or if it denotes a pathological process. The frequent comorbidities particularly present in the geriatric population that can affect kidney function, such as diabetes and cardiovascular diseases, amongst others, together with the increased prevalence of albuminuria in older subjects, and the recognition of glomerular sclerosis, tubular atrophy and vascular sclerosis in biopsy samples, represent further challenges to distinguish normal aging kidney function loss from pathological CKD. This is also important considering that CKD substantially increases the risk for cardiovascular and all-cause mortality, with low GFR correlating with worse outcome.

Since 2002, when the Kidney Disease Outcomes Quality Initiative (KDOQI) published a new chronic kidney disease (CKD) classification based on five categories of estimated glomerular filtration rate (eGFR), CKD has been defined as GFR <60 mL/min/1.73 m^2^ in the absence of kidney damage, as defined by structural of functional abnormalities (4). This threshold reflects a mean loss of 50% of kidney function in the healthy adult population. The KDIGO recommendations also define a GFR below 60 mL/min/1.73 m^2^ as a CKD, regardless of age (5). Although there is no precise definition of the normal range of GFR for older individuals, the use of this classification, with a fixed threshold for defining CKD based on eGFR values <60 mL/min/1.73 m^2^, does not consider the physiological GFR decline with aging and may result in an overdiagnosis of CKD in the elderly (6–8). In fact, an alternative age-adapted CKD definition, stratifying the GFR threshold according to age (75, 60, and 45 mL/min/1.73 m^2^ for ages younger than 40, 40–64, and 65 years or older, respectively) was proposed and analyzed in a large Canadian cohort, consisted of 127,132 individuals. Using a single, fixed eGFR threshold irrespective of age resulted in a 60% higher incidence of CKD. In this study, it is of note that 75% of individuals classified as CKD according to current fixed eGFR criteria <60 mL/min/1.73 m^2^ were 65 years or older and had an eGFR of 45–59 mL/min/1.73 m^2^ without significant albuminuria, raising the concern whether these individuals should be classified as CKD patients. The classification of older patients with age-related eGFR decline as having CKD is of crucial relevance, as it leads to an increase the risk of unnecessary interventions in much of this elderly population with additional implications for clinical practice and health policies (6–8).

Accurate assessment of renal function in the older population is of particular clinical significance for the diagnosis and classification of CKD, enabling tailored therapeutic interventions, and avoiding treatments of unnecessary and/or potential risk. GFR measurement can be carried out using conventional approaches through renal or plasma clearances of specific filtration markers, by measuring the marker concentration in plasma and urine or only in plasma, respectively (7). Alternatively, the use of eGFR is a more accessible approach for evaluating kidney function in a daily clinical practice. Several equations available have different performances characteristics concerning precision and accuracy and to avoid bias.

The present review is aimed at understanding the physiology of glomerular filtration in the elderly, highlighting the structural and functional changes associated with normal healthy aging leading to age related loss of kidney function. Also of interest is reviewing the different options to evaluate GFR in the elderly population to provide support to clinicians in the option and interpretation of the most appropriate method to either measure or estimate GFR.

## Glomerular filtration changes in the aging kidney

The kidneys receive 20–25% of the cardiac output. Appropriate glomerular filtration occurs to excrete waste products of metabolism and to maintain internal homeostasis of water and electrolytes. The most important physiological function of renal hemodynamics is to keep the blood flow and pressure within the glomerular capillary at levels that provide adequate filtration rate with subsequent optimum tubular reabsorption and secretion functions (9). An adequate glomerular filtration rate (GFR) depends on the maintenance of the structural glomerular filtration barrier such as fenestrated endothelium, glomerular basement membrane, and podocyte and slit diaphragm besides endothelial glycocalyx and specialized glycocalyx between the foot processes and basement membrane (10).

GFR is the best parameter for measuring kidney function. GFR represents the volume of plasma filtered by functioning nephrons over a specified period. Among normal adult individuals, GFR ranges from 100 to 125 mL/min/1.73 m^2^ adjusted to demographic parameters such as gender, race and age (11–17). The GFR adjustment for race is a subject of recent discussion, as reported elsewhere (18–20).

With the natural aging process, all organs, including the kidneys, are progressively affected by the decline in biological functions and progressive structural changes, regardless of the presence of disease. Indeed, in healthy individuals, age-related GFR decline has long been recognized. The first report on the decline of GFR with age, published in 1950, describes a decrease in GFR, measured by inulin clearance, from 123 ml/min/1.73 m^2^ to 65 ml/min/1.73 m^2^ at the age of 90, decreasing about 8 mL/min/1.73 m^2^ per decade (21, 22). The Baltimore Longitudinal Study of Aging (BLSA) reported a decrease in creatinine clearance of 0.75 mL/min/year in normal subjects aged 30–90 years followed up for a period of 14 years (23). The overall decline in GFR with age, analyzed by inulin clearance, starts at 30–40 years of age and becomes more prominent in adults older than 70 years (15, 24, 25).

Although the decrease in GFR with aging has been well-recognized, the exact estimation of the magnitude of the renal function decline with healthy aging is not yet well-established. Different methodological strategies, the use of endogenous or exogenous filtration markers to measure GFR, the equation applied to estimate GFR, the demographic representation of the healthy participants' population and the follow-up period represent issues that might account for discrepancies. Besides large geographical cohort and meta-analysis studies (11), evaluation of normal GFR has also been carried out with living kidney donor healthy individuals (12, 26–28).

Studies with living kidney donors confirmed a decrease in GFR with age, although with different decline rates. In analyzing GFR measured with ^51^Cr-EDTA in 241 subjects 40–73 years of age, Grewal et al. found that GFR decreased by 0.91 mL/min/1.73 m^2^ per year (29). Poggio et al., measuring GFR with ^125^I-iothalamate clearances in 1,057 donors, found a decline of 1.49 ± 0.61 ml/min/1.73 m^2^ per decade (12), whereas Rule et al., in a cross-sectional study including 1,203 adult living kidney donors, showed that GFR declined at a rate of 6.3 mL/min/1.73 m^2^ per decade (2). Pottel et al. measured the GFR of 633 potential living kidney donors (by inulin, iohexol, or ^51^Cr-EDTA) and confirmed a progressive decline with the age of an average of −0.89 ml/min/1.73 m^2^ per year, corresponding to a Full Age Spectrum (FAS) equation prediction of an average decline rate of −0.92 ml/min/1.73 m^2^ per year (30, 31).

More recently, the Renal Iohexol Clearance Survey in Tromsø 6 (RENIS-T6), a robust study including 1,594 healthy individuals from the general population (age 50–62 years) followed for more than 5 years, showed a measured GFR decline rate of 0.95 ± 2.23 ml/min/1.73 m^2^ per year (32). A meta-analysis of iohexol clearance measurements in three European population-based cohorts of individuals aged 50–97 showed that the mean GFR was lower by −0.72 mL/min/1.73 m^2^ per year for healthy men and −1.03 ml/min/1.73 m^2^ per year for unhealthy men, and −0.92 mL/ min/1.73 m^2^ per year for healthy women and −1.22 ml/min/1.73 m^2^ per year for unhealthy women (33). Table 1 summarizes the demographic characteristics, age ranges of study population, the corresponding changes of GFR, and methods used to measure GFR in the different mentioned studies.

**Table 1:** Demographic characteristics, age ranges of study population, the corresponding changes of GFR, methods used to measure GFR.

**References**	**Sample**	**Demographic** **characteristics**	**Age range** **(years)**	**Changes in GFR with** **age**	**Methods used** **to analyze** **GFR**
Davies et al. (21)	70 healthy individuals	100% male	24–89	GFR decline: from 122.8 to 65.3 mL/min/1.73 m^2^ (decreasing about 8 mL/min/1.73 m^2^ per decade)	Inulin clearance
Lindeman et al. (23)	254 healthy kidney subjects (non-proteinuric diabetes were included)	98% american predominately white	22–97	Creatinine clearence decline: 0.75 ml/min per year	Creatinine clearance
Fuiano et al. (24)	26 living kidney donors	100% males, young *n* = 15, older *n* = 11	19–32 65–76	GFR in young: 127 ± 5.8 mL/min/1.73 m^2^ GFR in older: 79 ± 4 mL/min/1.73 m^2^	Inulin clearance
Rule et al. (26)	365 living kidney donors	47.4% male, 80.3% white, age: 41.1 ± 11.4 yr	18–71	GFR decline: 4.9 mL/min/1.73 m^2^ per decade	Iothalamate clearance
Grewal et al. (29)	428 living kidney donors (241 aged > 40 yr)	49.1% male	40–73	GFR decline: 0.91 mL/min/1.73 m^2^ per decade	^51^Cr-EDTA clearance
Poggio et al. (12)	1,057 living kidney donors	44% male, 11% African American	38.5 ± 10.4	GFR decline: 1.49 ± 0.61 ml/min/1.73 m^2^ per decade	^125^I-Iothalamate clearance
Rule et al. (2)	1,203 adult living kidney donors	42% male, 93% white	18–77	GFR decline: 6.3 mL/min/1.73 m^2^ per decade	Iothalamate clearance
Kasiske et al. (27)	201 kidney donors, 203 paired controls	32% male	43.1 ± 11.9 43.4 ± 11.3	In kidney donors, GFR increased 1.47 ± 5.02 mL/min per year In controls, GFR declined 0.36 ± 7.55 mL/min per year	Iohexol
Baba et al. (28)	75,521 healthy individuals	47% male	42.8 ± 10.4	eGFR decline: 1.07 ± 0.42 ml/min/1.73 m^2^per year.	3-variable Japanese equation
Pottel et al. (30, 31)	633 living kidney donors	36.8% male	20–>70	GFR decline: 0.89 ml/min/1.73 m^2^ per year	Inulin, iohexol, or ^51^Cr-EDTA
Eriksen et al. (32)	1,594 healthy individuals from the general population	49% male 42% hypertension BMI:27.2 Kg/m^2^ UACR: 0.23 mg/mmol	50–62	GFR decline: 0.95 ± 2.23 ml/min/1.73 m^2^ per year	Iohexol clearance
Waas et al. (34)	13,381 individuals from a German population cohort	48.7% male 23.5% obese	35–74	eGFR decline: approximately 1 ml/min/1.73 m^2^ per year	eGFR calculated by CKD-EPI

In summary, a slow decline in GFR with age is expected in healthy individuals as shown in different geographical regions (11), with a median loss of eGFR per year of ~1 ml/min/1.73 m^2^ (34).

### Structural and functional changes in the aging kidney

The age-related decline in GFR is considered a physiological process after 30–40 years of age, with a more significant decline after the 70 s. With normal aging, nephron loss occurs and is detectable to some extent by the age-related decrease in GFR. Senescence causes functional and structural changes in the kidneys. Not only does the GFR decline with aging but so does the renal plasma flow, the glomerular capillary plasma flow rate, and the glomerular capillary ultrafiltration coefficient (Kf) (35–37).

#### Structural changes

Structural changes are associated with the senescence kidney process. With aging, the number of nephrons decreases among healthy adults (38–41). Under the age of 40, sclerotic glomeruli comprise <5% of the total, increasing thereafter, reaching as much as 30% of the glomerular population by the eighth decade, which can result in a progressive loss of 20–25% renal mass, mainly in the cortical region, in more advanced ages (42).

The loss of nephrons likely reflects a progressive degree of glomerulosclerosis, which increases with normal aging, even in healthy aging (2, 43–47). The accurate detection of the nephron number, which can only be assessed through histological analysis of kidney biopsies, is limited due to the invasive nature of this procedure. In this setting, the available information has been provided by studies carried out in the healthy kidney transplant donor population by means of biopsies obtained during the transplant surgery, enabling the analysis of kidney histomorphometry in different aged individuals (41, 48). Kidney biopsies obtained from the latter showed that nephron loss was characterized by a reduction in the total number of glomeruli at the expense of sclerotic glomeruli and non-sclerotic glomeruli. Evaluation of kidney cortical volume in this healthy population by imaging with computer tomography scans also identified a decrease in cortical volume (41). It is of note that the loss of nephrons with aging seems to be closely correlated not with the number of sclerotic glomeruli but, rather, with the decreased number of non-sclerotic glomeruli (41). A possible explanation for this apparent inconsistent discordance between nephron loss with aging and a relatively low detection of glomerulosclerosis relies on the theoretical concept that globally sclerotic glomeruli can be fully reabsorbed or may undergo changes such as atrophy and obsolescence (38). Hence, they can no longer be easily detected on tissue sections examined by light microscopy (41). This is of relevance due to the fact that the percentage of glomerulosclerosis routinely seen in kidney biopsy reports from elderly patients may substantially undervalue the true age-related loss of glomeruli. The process has not been fully elucidated yet, and further studies are needed to explain the pathophysiology of age-related renal dysfunction.

Along with the structural and functional impairment changes, an age-related decline in GFR occurs with healthy aging (48–51). Progressive loss of filtering glomeruli due to glomerulosclerosis or vanished glomeruli affects the remaining preserved nephrons to undergo compensatory hypertrophy responses (52, 53), with a compensatory increase in the single-nephron GFR and glomerular capillary hydraulic pressure aiming to preserve global GFR (48). However, the enlarged glomeruli derived from compensatory hypertrophy induce an increased tension in the glomerular capillary walls, leading to hypertension, hyperfiltration, and damage to the remaining nephrons (54). Experimental studies have also shown a reduction in Kf in older rats as a result of glomerular capillary permeability and the surface area available for filtration, and confirmed an increase in glomerular capillary hydraulic pressure due to the decrease in the afferent arteriolar resistance (36).

Increased glomerular volume associated with compensatory hypertrophy can induce podocyte injury and loss. Podocytes are crucial cells for maintaining the normal glomerular architecture and capillary permeability but, due to their terminal differentiation nature, they have a limited capacity to undergo cell division, regeneration, and repair (55). The degenerative aging phenotype along with the structural and functional changes that occur in the aging process, promote a greater podocyte detachment rate, with a decrease in the density of podocytes per glomerulus and ultimately capillary wrinkling, tuff collapse and periglomerular fibrosis (56).

**Figure 1:**
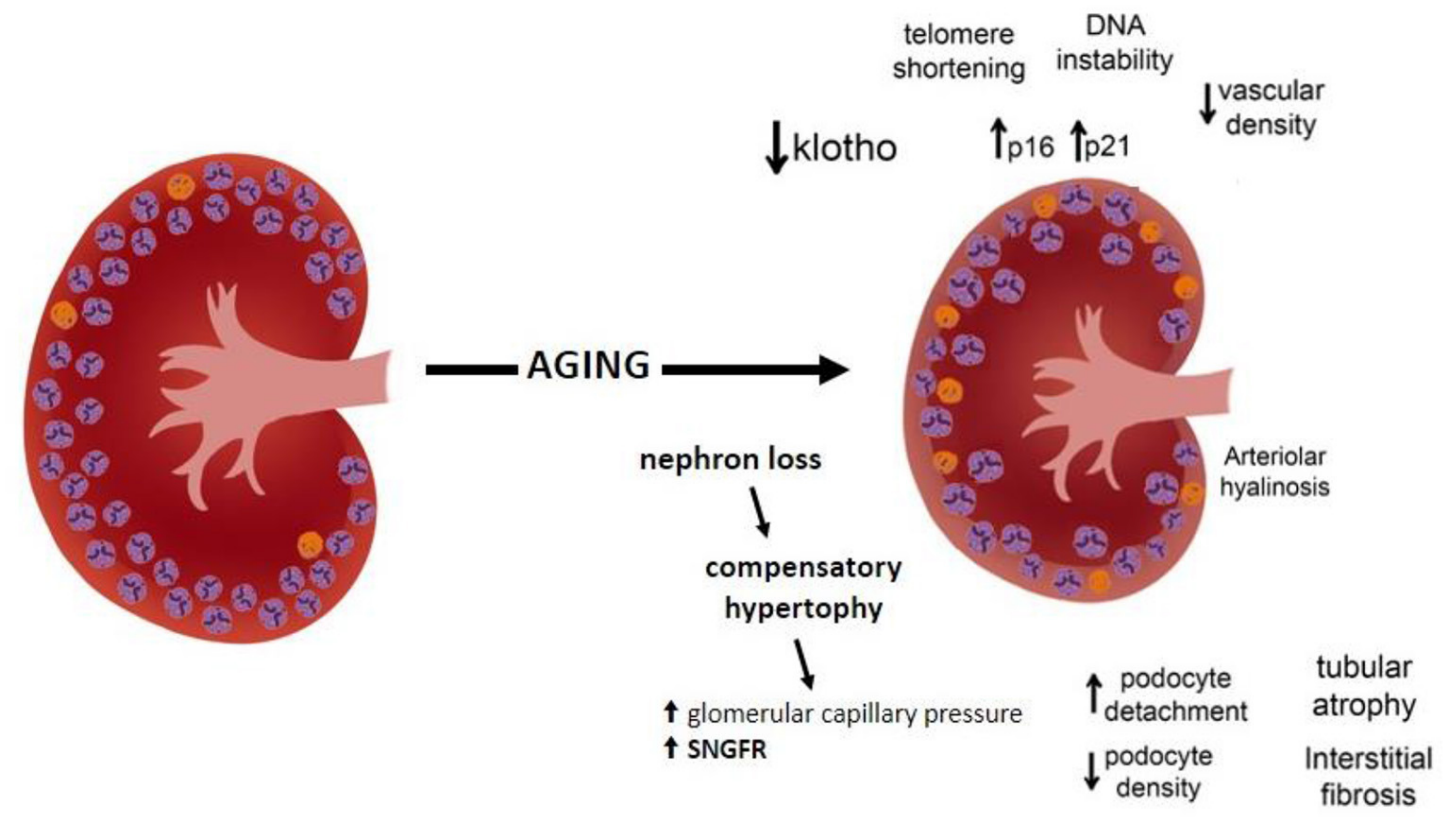
Schematic structural and functional changes in the aging kidney.

The pathophysiology of renal mass decrease may be related to several mechanisms, such as a decrease in klotho expression, an increase in telomere shortening, DNA instability, increased oxygen radicals, and overexpression of proteins that induce cell cycle arrest, with induction of p16 and p21 cell cycle inhibitors (57–59). Klotho, expressed predominantly in the kidney distal convoluted tubules, has been recognized as a key modulator of aging, with antiaging effects on several pathways. The klotho deficiency that occurs with the aging process accelerates aging-related diseases, including stroke, atherosclerosis, and osteoporosis. In contrast, overexpression of the gene encoding klotho extends lifespan in mice (60). In addition, the aging process increases the oxidant activity, decreases the autophagy, and enhances the capillary rarefaction contributing to the development of renal fibrosis (59) (Figure 1).

Besides glomerulosclerosis, other structural changes become more evident in the kidneys of elderly patients, including tubular atrophy, interstitial fibrosis, arteriolar hyalinosis, and atherosclerosis that increase with aging (2). One hypothesis is that fibrointimal hyperplasia of renal arterioles occurring with advanced age triggers glomerulosclerosis and consequent interstitial fibrosis and focal atrophy (61). Indeed, said age-related histological features are challenging to distinguish from disease-related renal pathological changes. The presence of comorbidities, abnormal urinary albumin excretion, and elevated blood pressure may weigh in favor of disease-related renal pathological changes. Finally, the number of renal cysts also increases with normal aging becoming very common in the kidney parenchyma (56).

Arterial hypertension, a high-sodium diet, the Western lifestyle, and its correlated comorbidities such as obesity and diabetes have already been associated with age-related loss of kidney function, although there is evidence that this process occurs despite the presence of these factors. Studies performed in patients without arterial hypertension or in diabetic patients showed similar declines in renal function rates (62, 63), suggesting not necessarily a pathological process.

#### Functional changes

Several studies indicate that renal hemodynamics is impaired with aging due primarily to a vascular process. The altered renovascular tone can be due to structural lesions in the renal vasculature, more evident in older patients with cardiovascular comorbidities, whilst pronounced renal vasoconstriction is also expressed in healthy normotensive elderly subjects, possibly due to functional abnormalities (24, 64, 65). With aging, there is compromised endothelium-dependent vasodilatation in the kidney characterized by a reduced vasodilatory response to stimuli such as acetylcholine, dopamine and nitric oxide, and greater sensitivity to vasoconstrictor stimuli, such as angiotensin, norepinephrine, and endothelin (24, 64, 66, 67). The tendency toward increased renal vasoconstriction due to a defect in nitric oxide-dependent response is also responsible for sodium retention (68) and the deregulation of the pressure-natriuresis response (69). In the elderly, the renin angiotensin system is suppressed. Although renin production and release are decreased with aging, leading to lower renin and aldosterone levels, the response to these hormones is exaggerated (36).

With the aging process, other changes in kidney function can be observed (Table 2). The ability to urinary concentration or dilution is impaired with advancing age and GFR deterioration. After fluid overload, older individuals have a decreased diluting capacity due possibly to a defect to generate free water. Older individuals also have a decreased ability for urinary concentration. Compared to younger individuals, older adults have about a 50% reduced capacity to conserve water and solutes under water deprivation. Reduced thirst response to osmotic changes, and changes in plasma osmolality, make elderly subjects more susceptible to developing disturbances in water homeostasis and volume depletion (42, 70). Decreased water-conserving capacity can have important clinical implications when older individuals do not have adequate access to water (70).

**Table 2:** Kidney functional changes with aging and declining GFR.

**Impairment in vasodilation response in renal hemodynamics**
• Greater sensitivity to vasoconstrictor stimuli (angiotensin, norepinephrine and endothelin). • Reduced vasodilatory response (acetylcholine, dopamine and nitric oxide).
**Decreased capacity to concentrate and dilute urine** • Urinary concentration Reduced capacity to conserve water and solutes under water deprivation. • Urinary dilution Decreased diluting capacity after fluid overload, possibly due to a defect to generate free water.
**Impaired renal regulation of sodium/potassium balance** • Sodium Lower capacity to reduce sodium excretion under sodium restriction. Lower capacity to excrete sodium under sodium load, leading to sodium retention and fluid volume expansion. • Potassium Lower capacity to decrease potassium excretion under high potassium diet
**Acid-base dysregulation** • Reduced capacity to buffer metabolic changes • Reduced capacity to excrete the excess H^+^ load and ammonium
**Calcium homeostasis** • Impaired vitamin D production in the skin • Impaired production of 1,25-(OH)2D3 • Increased PTH secretion • High FGF23 levels

Renal regulation of sodium/potassium balance is also impaired in the elderly. Individuals older than 60 years submitted to sodium restriction have a lower capacity to reduce sodium excretion. On the other hand, older individuals submitted to sodium load do not properly excrete sodium, leading to sodium retention and fluid volume expansion (42). Potassium homeostasis is also altered in aging. Animal studies demonstrated that potassium excretion was less efficient in aging rats fed with a high-potassium diet (71). In clinical settings, medications that interfere with potassium excretion should be carefully evaluated due to the propensity of older patients to develop hyperkalemia.

Aging people are also prone to developing acid-base dysregulation. The age-related decline in the GFR reduces the capacity of the kidney to buffer metabolic changes and excrete the excess H^+^ load and ammonium (72).

In addition, calcium homeostasis could be affected by aging. Older individuals present impaired vitamin D production in the skin, impaired production of 1,25-(OH) 2D3, and an age-related decrease in the capacity of 1,25-dihydroxyvitamin D3 to promote intestinal absorption of calcium, resulting in increased PTH secretion and higher FGF23 levels (73).

## How to evaluate and interpret GFR in the elderly

Several studies have addressed the discussion of the accuracy of GFR measurements in the elderly (15, 74–78). Conventional approaches for determining measured GFR use the principle of renal clearance of various substances, including endogenous filtration markers (creatinine or cystatin C) or exogenous markers (such as inulin, iohexol, iothalamate, or other radiolabeled compounds). These substances, excreted by the kidney, should ideally be exclusively excreted by glomerular filtration with no or minimal secretion by the tubules.

### GFR measurement using endogenous filtration markers

The determination of inulin clearance performed under continuous intravenous infusion and urine collection is considered the most accurate, and still the gold standard method for GFR measurement, but it is cumbersome, expensive and not feasible in daily clinical practice (79). Alternatively, renal clearance can be performed using creatinine, an endogenous product freely filtered by the glomerulus, though also secreted by tubular cells. Thus, urinary creatinine clearance, a simple and accessible method, remains a common conventional method used for renal function evaluation. However, in the elderly population, besides the potential errors associated with inadequate urine sample collection, the use of creatinine as a marker for determining GFR in this age group, characterized by progressive loss of muscle mass, represents an additional drawback. Creatinine is a nitrogenous substance derived from muscle metabolism. Reduction in muscle mass is a common aging-related condition associated with decreased appetite, decreased protein intake, reduced physical activity, and sarcopenia, causing not only reduction of muscle strength but also the loss of muscle mass, reflected as lower serum creatinine levels, which impact the creatinine-based GFR estimation. Therefore, the use of serum creatinine as a marker for estimating GFR in the elderly results in an overestimation of the renal function (80).

A disadvantage of urinary clearances for measuring GFR, particularly in the frail elderly population, is the troublesome related to urine sample collection, which can lead to errors due mainly to urinary losses and incomplete urinary bladder emptying, causing sampling errors that might jeopardize the results. In this setting, the evaluation of plasma clearances for measuring GFR provides more precise results than urinary clearances (77).

Cystatin C is a more reliable endogenous marker for the evaluation of renal function compared to creatinine. Cystatin is a substance constitutively produced by all nucleated cells. As a low molecular weight protein, cystatin is freely filtered by kidneys and catabolized in the tubules, where all metabolites are reabsorbed. Cystatin C is not dependent on muscle conditions (81) but is influenced by factors such as smoking, obesity, and inflammation. These features make this substance a more accurate alternative for measuring glomerular filtration.

### GFR measurement using exogenous filtration markers

Plasma clearances can also be performed using exogenous markers. The most widely used are the non-ionic contrast agent iohexol, particularly in Europe, and the iothalamate, more broadly used in the USA. Radiolabeled compounds such as ^51^Cr-EDTA, ^99m^Tc-DTPA, and ^125^I-iothalamate have also been employed for GFR measurements with different availability in different centers. Determination of plasma clearances comprises intravenous injection of the compound and subsequent blood-sample drawings at different time-points to construct a plasma disappearance curve of the exogenous marker. Although methods based on exogenous markers likely provide higher accuracy in determining GFR, they are usually more expensive and not widely accessible in routine practice. While they have also been considered as gold standard for GFR determination in many studies, it is important to be aware that substances such as iothalamate are secreted by renal tubules and therefore, can overestimate GFR as compared to inulin clearances (79). Notwithstanding these considerations, plasma clearances are considered accurate methods of measuring GFR.

### GFR estimation using equations

In clinical settings, an alternative to provide reliable and easy evaluation of kidney function in the elderly is the use of estimated GFR (eGFR) equations rather than direct measurement of GFR. eGFR based on equations is a simple and less expensive assessment, recommended by clinical guidelines from the National Kidney Foundation (NKF KDOQI) and Kidney Disease Improving Global Outcomes (KDIGO) (5, 82). Different formulas derived from large cohorts have been developed to estimate GFR in adults, based on serum creatinine or cystatin C (18, 83, 84).

The Cockcroft-Gault equation was the first equation developed for estimating creatinine clearance. In 1999, Levey and coworkers developed the first equation to estimate GFR, the Modification of Diet in Renal Disease (MDRD), normalized to 1.73 m^2^ body surface area (BSA) derived from measured iothalamate-measured GFR (83). In both equations, however, the geriatric population was underrepresented. In addition, these equations are based on serum creatinine, with the concerns for older patients already addressed. Both equations have been widely worldwide used, but they usually overestimate measured GFR in older patients (77, 78, 85), although Cockcroft-Gault adjusted for BSA yields a smaller bias (77). Of note, BSA-GFR is not a good normalization index in frail patients. In such a case, a BSA-GFR is usually overestimated, impairing the fair evaluation and clinical management.

The Chronic Kidney Disease-Epidemiology Collaboration equation (CKD-EPI) (18), also developed by Levey et al., is the most common currently used equation in routine clinical practice. Although CKD-EPI was developed to improve the estimation of GFR in older patients, participants older than 65 years of age were less represented in the CKD-EPI study population (only 13% of the whole population). Compared to iohexol plasma clearance, creatinine based CKD-EPI (CKD-EPICr) in older patients also overestimates GFR (77).

Equations based on serum cystatin C levels have proved their superiority over creatinine-based formulas in the elderly (86–88). A meta-analysis of 46 cross-sectional studies confirmed that the accuracy of cystatin C is superior to creatinine-based equations (eGFRCr) (34, 78, 89–91). Alternatively, equations based on the combination of cystatin C with creatinine (CKD-EPICr+Cys) have been developed, with even better precision and accuracy compared to equations based on creatinine alone or cystatin C alone (77, 92, 93). In fact, current guidelines, besides recommending CKD-EPI equations for estimating glomerular filtration, recommend the combination of creatinine and cystatin C (eGFRCr+Cys) as a more accurate approach (5).

To provide a more precise and accurate GFR evaluation in the elderly, the Berlin Initiative Study (BIS) analyzed a cohort of subjects aged over 70 years (77), resulting in the development of two equations designed for older individuals: the BIS1, a creatinine-based equation, and the BIS2, a creatinine and cystatin C–based equation. Both equations provided very good agreements with measured GFR, including in Chinese older subjects (94).

Other eGFR equations for the elderly population have been described such as the Caucasian, Asian, Pediatric and Adult (CAPA) (95), the Japanese equations (96, 97), in a country where the population of persons older than 65 years reached more than 25% of the total population, and the Lund-Malmö revised creatinine equation (LMRCr), developed in a Swedish cohort population population (98). LMRCr showed better performances than CKD-EPIcr. Additionally, combining LMRCr with the CAPA cystatin C equation (CAPACys), by performing the arithmetic mean of the LMRCr and CAPA equations (MEANLMR+CAPA), improved the accuracy of the GFR estimation (99).

More recently, in 2016, Pottel et al. developed, in European healthy individuals, a new eGFR equation for the elderly population, the FAS (100), based on serum creatinine. The validation was carried out comparing with measured GFR (inulin, iohexol, and iothalamate clearance). The FAS equation improved the precision and accuracy of eGFR, especially in older adults, also in chinese older individuals (101). Subsequently, besides the Full Age Spectrum creatinine equation (FASCr), its combination with cystatin C (FASCr+Cys) was proposed (31).

Table 3 summarizes the main studies evaluating eGFR against a gold-standard method to measure GFR.

**Table 3:** Summary of studies addressing equations to estimate glomerular filtration rate and participation of older individuals.

**References**	**eGFR equation**	**Sample size**	**Gold-standard**	**Conclusion**
Levey et al. (83)	MDRD	*n* = 1,628 *n* = 681 >55 yr *n* = 0 >70 yr	^125^I-iothalamate	MDRD: more accurate than measured creatinine clearance (overestimates GFR by 19%) and Cockcroft-Gault formula (overestimates GFR by 16%).
Levey et al. (83)	CKD-EPI	*n* = 8,254 *n* = 69 >75 yr	^125^I-iothalamate	CKD-EPI: more accurate than MDRD.
Schaeffner et al. (77)	BIS 1 (creatinine) BIS 2 (cystatin C)	*n* = 610 ≥70 years	Iohexol	BIS 2: lowest bias and smallest misclassification rate; BIS 1: smallest misclassification rate among the creatinine-based equations.
Grubb et al. (95)	CAPA	*n* = 1,200 from a Swedish cohort and *n* = 413 from a Japanese cohort	Inulin	Substandard P30 among the elderly (>80 years old)
Björk et al. (98)	LMR	*n* = 850 adults	Iohexol	Increased accuracy at measured GFR ≥90 mL/min/1.73 m^2^ The LM equations cannot be recommended for use in general clinical practice
Pottel et al. (100)	FAS	*n* = 6,870 *n* = 1,774 ≥70 yr	Inulin, iohexol and iothalamate	Less biased and more accurate than CKD-EPI for older adults.

MDRD, Modification of Diet in Renal Disease; CKD-EPI, Chronic Kidney Disease-Epidemiology Collaboration; BIS, Berlin Initiative Study; CAPA, Caucasian, Asian, Pediatric and Adult; LMR, Lund-Malmö revised creatinine equation; FAS, Full Age Spectrum.

## For the geriatric population, which equation is more accurate to estimate GFR?

Compared to gold standard clearance methods, the currently available equations for determining eGFR have different performances related to bias, precision, and accuracy, which highlights the difficulty in defining an accurate estimation of GFR in older patients. It is important to emphasize that the Cockcroft-Gault equation is not similar to CKD-EPI or other eGFR equations.

Table 4 summarizes the results of studies comparing discrepancies of GFR estimating equations in older adults examining performances between the eGFR analyzed by metrics such as bias, precision, and accuracy. Bias represents the median difference between measured GFR and estimated GFR. It is important to identify the eGFR equation with the smallest bias. Bias with negative values indicates that the equation overestimates the GFR. Precision also represents the differences between measured GFR and estimated GFR but it is expressed as the interquartile range (IQR). Accuracy can be evaluated as P30, which represents the percentage of estimates within 30% of the measured value. The highest P30 values reflect better accuracy of eGFR compared to measured GFR.

**Table 4:** Studies comparing discrepancies of GFR estimating equations and estimated creatinine clearance (Cockcroft–Gault) in older adults examining performances between the eGFR analyzed by metrics such as bias, precision, and accuracy.

	**Schaeffner et al. (77)**	**Bjork et al. (92)**	**Bjork et al. (93)**	**Selistre et al. (102)**
**(A) Performances between the eGFR analyzed by median bias**.				
*n*	610	3,226	805	2,247
Age (yr)	>70	70–89	74–93	65–90
Cohort	The BIS cohort	5 cohorts (Caucasian)	AGES-Reykjavik elderly cohort	Single center French cohort
Measured GFR	Plasma iohexol clearance	Plasma iohexol clearance	Plasma iohexol clearance	Inulin clearance
Cockcroft–Gault Cr	2.53 (−4.06 to 9.21)	n.a	n.a	n.a
MDRDCr	11.29 (3.85–17.68)	n.a	n.a	n.a
CKD-EPICr	9.69 (2.45–15.49)	3.6 (3.2–4.0)	2.7 (2.1–3.3)	−2.0 (−3.0 to −1.0)
BIS-1 (Cr)	0.80 (−5.03 to 6.11)	1.7 (1.2–2.0)	n.a.	−2.0 (−3.5 to −1.5)
LMRCr	n.a	−0.7 (−1.0 to −0.4)	−4.8 (−5.4 to −4.2)^a^	2.0 (1.5–3.5)
FASCr	n.a	0.6 (0.3–0.9)	−5.7 (−6.3 to −5.1) ^a^	0.0 (−0.5 to 0.5)
CKD-EPICys	2.05 (−3.23 to 8.61)	−2.7 (−3.1 to −2.3)	n.a	n.a
FASCys	n.a	−1.1 (−1.6 to −0.8)	n.a	n.a
CAPA (Cys)	n.a	−1.4 (−1.8 to −1.0)	n.a	n.a
CKD-EPICr+Cys	n.a	−0.1 (−0.4 to 0.2)	0.6 (−0.1 to 1.2)	n.a
BIS-2 (Cr+Cys)	0.87 (−4.40 to 4.98)	−1.2 (−1.5 to −0.8)	n.a	n.a
MEANLMR+CAPA	n.a	−1.0 (−1.3 to −0.6)	−2.7 (−3.2 to −2.1) ^a^	n.a
FASCr+Cys	n.a	−0.8 (−1.1 to −0.5)	−5.9 (−6.5 to −5.4) ^a^	n.a
**(B) Performances between the eGFR analyzed by IQR–Precision**.				
CKD-EPICr	n.a	12.3 (11.9–13.0)	12.1 (11.2–13.4)	15.0 (14.5–17.0)
BIS-1 (Cr)	n.a	11.6 (11.1–12.1)	n.a	15.0 (14.0–16.5)
LMRCr	n.a	10.5 (10.1–11.0)	10.8 (10.1–11.5)^b^	14.0 (13.0–15.5)
FASCr	n.a	11.1 (10.6–11.5)	10.7 (9.9–11.9)^b^	14.0 (12.5–15.0)
CKD-EPICys	n.a	11.8 (11.3–12.5)	n.a	n.a
FASCys	n.a	12.2 (11.7–12.8)	n.a	n.a
CAPA (Cys)	n.a	11.9 (11.3–12.6)	n.a	n.a
CKD-EPICr+Cys	n.a	10.2 (9.6–10.8)	10.2 (9.0–11.1)	n.a
BIS-2 (Cr+Cys)	n.a	10.5 (10.0–11.0)	n.a	n.a
MEANLMR+CAPA	n.a	9.2 (8.8–9.6)	9.3 (8.5–10.1)^c^	n.a
FASCr+Cys	n.a	10.1 (9.7–10.7)	10.0 (9.1–10.9)^c^	n.a
**(C) Performances between the eGFR analyzed by P30 accuracy**.				
Cockcroft–GaultCr	87.4	n.a	n.a	n.a
MDRDCr	70.9	n.a	n.a	n.a
CKD-EPICr	77.9	76.4 (74.9–77.9)	91.7 (89.9–93.4)	77.0 (73.0–80.0)
BIS-1 (Cr)	95.1	77.5 (76.1–78.9)	n.a.	76.0 (72.5–79.5)
LMRCr	n.a	83.5 (82.2–84.8)	95.0 (93.5–96.5)^b^	80.0 (76.5–83.5)
FASCr	n.a	80.9 (79.5–82.3)	95.8 (94.4–97.2)^b^	78.5 (75.0–82.0)
CKD-EPICys	89.1	78.8 (77.3–80.4)	n.a	n.a
FASCys	n.a	80.9 (79.4–82.4)	n.a	n.a
CAPA (Cys)	n.a	80.3 (78.8–81.8)	n.a	n.a
CKD-EPICr+Cys		86.8 (85.5–88.1)	96.1 (94.8–97.4)	n.a
BIS-2 (Cr+Cys)	96.1	85.7 (84.4–87.0)	n.a	n.a
MEANLMR+CAPA	n.a	88.7 (87.5–89.9)	97.3 (96.2–98.4)^b^	n.a
FASCr+Cys	n.a	85.7 (84.4–87.1)	97.8 (96.7–98.8)^b^	n.a

a Significantly worse (*P* < 0.05) than corresponding CKD-EPI equation.

b Significantly better (*P* < 0.05) than corresponding CKD-EPI equation.

c No statistical difference (*P* < 0.05) compared with corresponding CKD-EPI equation.

Schaeffner and coworkers compared performances of the eGFR equations, consisting of the BS1 and BS2 equations, Cockcroft-Gault, MDRD, CKD-EPICr, and CKD-EPICys, with the gold standard iohexol plasma clearance measurement in an elderly population-based cohort [the Berlin Initiative Study (BIS)] (77). BIS equations presented the smallest median bias compared with Cockcroft–Gault, MDRD, and CKD-EPICr equations, which have a higher median bias that overestimates GFR. Accordingly, the P30 values were highest for the BIS equations, followed by the CKD-EPICys and Cockcroft–Gault equations.

The 2017 study of Bjork, a large multicenter cohort of Europeans that enrolled 3,226 individuals 74–93 years of age, validated four creatinine equations (the CKD-EPICr, BIS1Cr, LMRCr, and FASCr) compared to iohexol plasma clearance as a gold-standard method (92). LMRCr had the most stable performance compared to measured GFR. FASCr can also be a good alternative to CKD-EPICr in creatinine based estimation of GFR. The addition of cystatin C to the equations (BIS2Cr+Cys, CKD-EPICr+Cys, FASCr+Cys, and MEANLMR+CAPA) improved the performances, particularly P30 accuracy.

In 2018, Björk et al. compared the performance of GFR estimating equations using creatinine and cystatin C in a cohort of elderly patients from Iceland who participated in the AGES-Reykjavik Study (93). A subgroup of 805 Caucasian individuals, 74–93 years of age, was enrolled in the period between 2007 and 2011 to participate in a substudy that measured GFR using plasma iohexol clearance (the AGES-Kidney Study). Different GFR equations such as LMRCr MEANLMR+CAPA, FASCr, and FASCr+Cys were compared to CKD-EPICr and CKD-EPICr+Cys, All equations showed high accuracy (P30 >90%). Although there were differences in performance, none of the creatinine-based equations was clearly superior in this cohort of older individuals. However, the addition of cystatin C in creatinine-based equations improved the performance of the equations. Fan et al. (103) also analyzed the 805 participants of this AGES-Reykjavik Study, comparing the performance of the Japanese, BIS, and CAPA equations with that of the CKD-EPI equations. They concluded that none of the Japanese, BIS, and CAPA equations were superior to the CKD-EPI equations. The addition of cystatin C in the CKD-EPIcr equation improved the performance of CKD-EPICr and CKD-EPICys.

Selistre et al., in a large single-center French population (*n* = 2,247) aged 65–90 years with various degrees of kidney impairment, tested four different equations (CKD-EPI, BIS-1, LMR, and FAS) compared to measured GFR using inulin clearance (102). The authors tested the bias, precision, and accuracy of these equations and found that the performance of the CKD-EPI equation was not different from that of BIS-1, LMR and FAS. In patients 75 years or older with measured GFR <45 ml/min/1,73 m^2^, LMR and BIS were more accurate than CKD-EPI and FAS but none of them had a superior diagnostic performance.

Recently, Yamaguchi et al. estimated GFR based both on serum creatinine and cystatin C in 19,764 individuals aged 18–103 years (mean 77.0 years) separated into two groups, <75 and >75 years old (the older group, *n* = 12,518, mean age 82.8 years old). The analysis of discrepancies between the CKD-EPI, Japanese, and BIS equations showed that eGFRcr was overestimated with CKD-EPI or the Japanese equation. They concluded that estimation of GFR using serum cystatin C provided more accuracy in elderly people (78).

It is worth mentioning that measured GFR is considered the gold-standard approach to access GFR. This is important because any equation used to estimate GFR may have an average error as wide as 30% or even larger, even while choosing that with higher accuracy, it has a bias that may reach 30 ml/min of difference from the measured filtration rate (104). Therefore, this should be kept in mind when interpreting studies.

## Clinical management of age-related GFR changes in the elderly

Accurate determination of GFR in the older population is of crucial importance for the correct evaluation of age-related kidney dysfunction, which implies appropriate management related to the stage of kidney disease.

In clinical practice, one of the most important aspects to be considered is the adequate adjustment of the dosage of prescribed drugs to avoid overdosing. Elderly patients have often been submitted to polypharmacy and many of these medications have renal excretion, which requires dose reductions adjusted to the degree of GFR compromised, more easily calculated by the GFR equations. Whether it is a pathological or age-related reduction in GFR, the dosage of renal-eliminating drugs should be adjusted according to the GFR, to avoid potential harm.

A great number of older individuals suffer chronic and acute pain, with a particularly high prevalence of joint pains and neuralgia. Non-steroidal anti-inflammatory drugs (NSAIDs) are often prescribed for the management of pain relief in the elderly population. NSAIDs are considered nephrotoxic drugs, with the exposure associated with a high risk of GFR decline of ≥30% in individuals with eGFR <60 ml/min/1.73 m^2^ (105). Hence, the prescription of NSAIDs should be done carefully in this population.

The use of iodinated contrast media is another potential cause of kidney injury possibly leading to kidney adverse outcomes such as renal dysfunction characterized by abrupt GFR decline and, eventually, the need for dialysis. The use of iodine-based contrast media for diagnostic and interventional radiology for the geriatric population has exponentially increased in the last decades, in oncological settings, cardiovascular imaging examinations, and other clinical situations. Risk factors for developing contrast nephrotoxicity include pre-existing CKD, dehydration, diabetes, and the use of ACE inhibitors, diuretics, and NSAIDs, all common situations in older patients providing high susceptibility to developing worsening kidney function (106, 107). The advent of iso-osmolar and hypo-osmolar agents decreased the incidence of contrast-induced AKI. Thus, the decision on its use must be considered, evaluating the risk-benefit of the imaging examination. Prehydration and n-acetylcysteine, traditionally used as protective measures, have been questioned since the latest clinical trials and have not shown benefits in their use (108, 109).

Adjustment of drug dosing in the elderly according to GFR is widely employed in clinical settings important for avoiding harmful complications. In the aging population, type 2 diabetes is a prevalent condition, and for instance, the risk of hypoglycemia can be avoided, at least in part, by the adjustment of oral antidiabetic agents. Older patients are often affected by infections for which antimicrobials are prescribed. Therefore, the knowledge of GFR is crucial in determining the ideal dose adjustment. In situations of a moderate/severe reduction in GFR, attention must be paid to avoid toxicity, and the risk of worsening renal function is needed.

In summary, the clinical management of older individuals should be based on “do not harm.” The use of contrast should be leveraged according to each indication. Besides antimicrobials, all prescriptions for older individuals should consider possible the risk of further reduction in GFR. Adjustment according to measured or estimated GFR is advised. All patients should be alert against the use of potential renal-damage drugs.

## Conclusion

The population is aging. With the natural aging process, all organs, including the kidneys, undergo aging-related changes characterized by a progressively decline in biological functions and anatomical changes. The loss of kidney function due to advancing age is expressed by GFR decline. Accurate assessment of renal function in the elderly is of particular clinical importance for the appropriate evaluation and management of age-related kidney dysfunction.

The gold standard methods to measure GFR include inulin clearance and plasma clearances based on clearance of radiolabelled or non-radiolabelled exogenous markers, which are laborious, expensive and not feasible in daily clinical practice. In clinical settings, an alternative to gold standard methods still capable of easily provide reliable evaluation of kidney function in the elderly is the use of estimated GFR (eGFR) equations. Hitherto, no equation precisely mirrors the measured GFR in older patients, and therefore, the results should be meticulously interpreted. The currently available equations for determining eGFR have different performances that lead to important discrepancies even in equations specifically developed for older patients. New techniques to measure GFR should be the target of future studies. The ideal method should be cost-effective and not rely only upon serum creatinine, particularly in the elderly and frail patients with sarcopenia and loss of muscle mass. Until then, equations should be used based on either creatinine or preferentially cystatin C, when available. So far, the CKD-EPI seems to be the best choice.

## Author contributions

IN planned the review article and drafted the manuscript. GS-C, LA, and RE helped to draft the manuscript. RE, LA, VAC, WJ-F, and IN gave important intellectual contributions. All authors read and approved the final version.

## Conflict of interest

The authors declare that the research was conducted in the absence of any commercial or financial relationships that could be construed as a potential conflict of interest.

## Publisher's note

All claims expressed in this article are solely those of the authors and do not necessarily represent those of their affiliated organizations, or those of the publisher, the editors and the reviewers. Any product that may be evaluated in this article, or claim that may be made by its manufacturer, is not guaranteed or endorsed by the publisher.
